# Constitutive Cytomorphologic Features of Medullary Thyroid Carcinoma Using Different Staining Methods

**DOI:** 10.3390/diagnostics11081396

**Published:** 2021-08-02

**Authors:** Chih-Yi Liu, Chien-Chin Chen, Andrey Bychkov, Shipra Agarwal, Yun Zhu, Jen-Fan Hang, Chiung-Ru Lai, Hee Young Na, So Yeon Park, Weiwei Li, Zhiyan Liu, Deepali Jain, Ayana Suzuki, Mitsuyoshi Hirokawa, Noel Chia, Min En Nga, Tikamporn Jitpasutham, Somboon Keelawat, Shinya Satoh, Dilini Gunawardena, Priyanthi Kumarasinghe, Chan Kwon Jung, Kennichi Kakudo

**Affiliations:** 1 Division of Pathology, Sijhih Cathay General Hospital, New Taipei City 221, Taiwan; 2 School of Medicine, College of Medicine, Fu Jen Catholic University, New Taipei City 221, Taiwan; 3 Department of Pathology, Ditmanson Medical Foundation Chia-Yi Christian Hospital, Chiayi 600, Taiwan; hlmarkc@gmail.com; 4 Department of Cosmetic Science, Chia Nan University of Pharmacy and Science, Tainan 717, Taiwan; 5 Department of Pathology, Kameda Medical Center, Kamogawa, Chiba 296-8602, Japan; bychkov.andrey@kameda.jp; 6 Department of Pathology, Nagasaki University Graduate School of Biomedical Sciences, Nagasaki 852-8523, Japan; 7 Department of Pathology, All India Institute of Medical Sciences, New Delhi 110029, India; drshipra0902@gmail.com (S.A.); deepalijain76@gmail.com (D.J.); 8 Department of Pathology, Jiangsu Institution of Nuclear Medicine, Wuxi 214063, China; zhuyun@jsinm.org; 9 Department of Pathology and Laboratory Medicine, Taipei Veterans General Hospital, Taipei 112, Taiwan; jfhang@vghtpe.gov.tw (J.-F.H.); crlai@vghtpe.gov.tw (C.-R.L.); 10 School of Medicine, National Yang Ming Chiao Tung University, Taipei 112, Taiwan; 11 Department of Pathology, Seoul National University Bundang Hospital, Seoul National University College of Medicine, Seongnam 13620, Korea; 0614nina@hanmail.net (H.Y.N.); sypmd@snu.ac.kr (S.Y.P.); 12 Department of Pathology, Shandong University School of Basic Medical Sciences, Jinan 250012, China; liweiweizhao@163.com; 13 Department of Pathology, Shanghai Jiao Tong University Affiliated Sixth People’s Hospital, Shanghai 200233, China; zhiyanliu@sdu.edu.cn; 14 Department of Diagnostic Pathology and Cytology, Kuma Hospital, Kobe 650-0011, Japan; suzuki01@kuma-h.or.jp (A.S.); mhirokawa@kuma-h.or.jp (M.H.); 15 Department of Pathology, National University Hospital, Singapore 119074, Singapore; noelcyd@gmail.com (N.C.); patngame@nus.edu.sg (M.E.N.); 16 Department of Pathology, Faculty of Medicine, Chulalongkorn University, Bangkok 10330, Thailand; jane_bt@hotmail.com (T.J.); Somboon.Ke@chula.ac.th (S.K.); 17 Department of Endocrine Surgery, Yamashita Thyroid and Parathyroid Clinic, Fukuoka 812-0034, Japan; shinya.satoh.48128@gmail.com; 18 School of Pathology & Laboratory Medicine, University of Western Australia, Perth, WA 6009, Australia; dilini.gunawardena@health.wa.gov.au (D.G.); Priyanthi.Kumarasinghe@health.wa.gov.au (P.K.); 19 Department of Hospital Pathology, College of Medicine, The Catholic University of Korea, Seoul 06591, Korea; ckjung@catholic.ac.kr; 20 Department of Pathology and Thyroid Disease Center, Izumi City General Hospital, Izumi, Osaka 594-0073, Japan; kakudo@thyroid.jp

**Keywords:** cytology, fine-needle aspiration, medullary thyroid carcinoma, sensitivity, specificity, thyroid

## Abstract

(1) Background: Accurate preoperative identification of medullary thyroid carcinoma (MTC) is challenging due to a spectrum of cytomorphologic features. However, there is a scarcity of studies describing the cytomorphologic features as seen on fine-needle aspiration (FNA) smears prepared using different staining methods. (2) Methods: We performed a retrospective study on MTC cases with available FNA slides from 13 hospitals distributed across 8 Asia-Pacific countries. The differences in the constitutive cytomorphologic features of MTC with each cytopreparatory method were recorded. A comparative analysis of cytologic characteristics was carried out with appropriate statistical tests. (3) Results: Of a total of 167 MTC samples retrospectively recruited, 148 (88.6%) were interpreted as MTC/suspicious for MTC (S-MTC). The staining methods used were Papanicolaou, hematoxylin-eosin, and Romanowsky stains. Seven out of the eleven cytologic criteria can be readily recognized by all three cytopreparatory methods: high cellularity, cellular pleomorphism, plasmacytoid cells, round cells, dyshesive cells, salt-and-pepper chromatin, and binucleation or multinucleation. An accurate diagnosis was achieved in 125 (84.5%) of the 148 samples whose FNAs exhibited five or more atypical features. Conclusions: The present work is the first study on MTC to compare the morphological differences among the cytologic staining techniques. We investigated the constitutive features and the reliability of diagnostic parameters. A feasible scoring system based upon cytomorphologic data alone is proposed to achieve a high degree of diagnostic accuracy.

## 1. Introduction

Medullary thyroid carcinoma (MTC) is a rare neuroendocrine carcinoma, which constitutes less than 2–3% of all thyroid malignancies [1]. Fine-needle aspiration (FNA) with ultrasound imaging is the first-line modality for the diagnosis of MTC. However, on aspiration cytology, the diagnostic accuracy for MTC is less consistent than for the more frequent, follicular cell-derived thyroid tumors [2]. The diverse appearances of MTC pose diagnostic difficulties due to morphologic overlap with other thyroid tumors. In previous studies, the reported diagnostic accuracy of MTC on FNA ranged from 12.5% to 100.0% [3,4,5,6,7,8]. In their meta-analysis of 641 MTCs, Trimboli et al. reported an overall sensitivity of only 56.4% [5]. Our recent report depicted that the diagnostic rate was increased from 68.3% to 91.7%, after detailed review and exceptional attention to cytologic features diagnostic for MTC. Recognition of those cytomorphologic clues can help improve diagnosis sensitivity.

The Bethesda System for Reporting Thyroid Cytopathology (TBSRTC) was established to standardize reporting and cytologic criteria in aspiration smears [9]. If the cytopathologic features raise the possibility of MTC, the diagnosis of TBSRTC category V (suspicious for malignancy/suspicious for MTC) or category VI (malignant, MTC) is used [9]. Moreover, the combined use of immunocytochemical staining (ICC) and/or calcitonin measurement in FNA washout fluid has demonstrated great diagnostic benefits [4,10,11,12,13]. Our previous survey found that the ancillary tests are not routinely adopted in Asian thyroid cytology practice at the time of the survey [14]. Thus far, the utility of concurrent calcitonin measurement and ICC has been noted in Japan and Australia. The cytology practice in Japan, China, and Australia includes calcitonin measurement in FNA washout fluid. In Asian countries, FNA alone is likely to be used for the initial workup for MTC and can cause a diagnostic dilemma without prompt ancillary tests [14]. Our previous study also found that different countries used different preparation methods and stains for FNA samples [14].

Clinically, the management of patients with indeterminate FNA specimens is variable, and the extent of surgery is often dependent upon how the FNA report is phrased. The cytologic interpretation is likely to be dependent upon the observer’s experience and also affected by the intrinsic limitations of the technique. To the best of our knowledge, no study has compared the differences in the cytomorphology of MTC using conventional staining methods. Therefore, the present study aimed to identify the pitfalls and intrinsic limitations of each staining method. To further improve the diagnostic performance, we assessed all the cytologic features that might have been responsible for misinterpretation. This study also proposes a low threshold to either perform or suggest further testing in thyroid nodules suspicious for MTC on aspiration cytology. 

## 2. Materials and Methods

### 2.1. Study Design and Sample Population

This study was performed within the network of the Asian Working Group in Thyroid Pathology [15]. Twenty-three cytopathologists from 13 institutions of 8 Asia-Pacific countries participated in this study. Local databases were searched retrospectively for histopathologically confirmed cases of MTC over a period ranging from 10 to 20 years. Cases with available preoperative FNA slides were enrolled. Either histological or cytopathological slides were confirmed and re-evaluated independently.

In our previous study [14], among 145 patients with histologically confirmed MTC, conventional smear samples were prepared in 123 cases, liquid-based samples were collected from 13 cases, and 9 cases had both preparations. We retrospectively observed that the overall diagnostic accuracy in detecting MTC was consistently high using Romanowsky stain, Papanicolaou stain, and/or hematoxylin and eosin (H&E) stain. Integration with ancillary tests achieved a cytologic diagnosis of MTC with excellent accuracy.

In the previous report, we noticed that the cellular heterogeneity of MTCs might explain the misinterpretation based solely on traditional cytologic methods. In the current study, we aimed to focus on different study objectives and analyzing strategies to assess the cytomorphologic features that contributed to diagnostic discrepancies. We enrolled MTC patients with available conventional smears. Details regarding staining methods (Papanicolaou, Romanowsky, and H&E), cytologic diagnoses, and cytomorphologic parameters were recorded. The cytologic materials consisted of one or two types of stains for each patient. As a result, a total of 168 cytopathology samples from 132 patients with MTC were obtained for this new study proposal (13 cases with liquid-based samples were excluded due to morphological disparity).

### 2.2. Cytopathologic Evaluation

All cytology preparations from histologically proven MTC cases were retrieved from the archives and reviewed. The smears were categorized as per TBSRTC into unsatisfactory, benign, atypia of undetermined significance or follicular lesion of undetermined significance (AUS/FLUS), follicular neoplasm, or suspicious for a follicular neoplasm (FN/SFN), suspicious for malignancy, and malignant [9]. The interpretation as “follicular neoplasm, Hürthle cell type” (FN-H) was merged into a diagnostic category, “FN/SFN.”

The diagnostic cytomorphologic criteria of MTC included moderate to marked cellularity, isolated cells alternate with syncytium-like clusters, polymorphous cell populations (plasmacytoid, polygonal, round, and/or spindle-shaped), mild to moderate nuclear pleomorphism, and features of neuroendocrine differentiation, such as round, oval, or eccentrically placed nuclei, with “salt-and-pepper” chromatin, binucleation, granular cytoplasm, and background amyloid [8,13]. The malignancy not otherwise specified (NOS) cytologic diagnosis indicated that an exact classification could not be determined cytologically. The suspicious cytology group included suspicious for MTC (MTC-S). Concordance between cytology and histology was considered only if cytologic features were suspicious or diagnostic for MTC. The cytologic samples were sorted into three groups based on the staining methods: Papanicolaou stain, Romanowsky stain, and H&E stain.

### 2.3. Comparison of Cytomorphologic Features

The cytomorphologic features of MTC in different diagnostic groups (accurately categorized and discordant), as well as staining methods (Papanicolaou stain, Romanowsky stain, and H&E stain), were analyzed. The key factors encompassed nuclear and cytoplasmic features of MTC, as well as background characteristics among the different groups.

The presence/absence of the following parameters was recorded in all the included cohorts: (1) cellular aspirate (i.e., encompassed moderate to marked cellularity); (2) cellular pleomorphism; (3) cell populations (plasmacytoid, round, polygonal, and spindle-shaped); (4) dyshesive cells; (5) “salt-and-pepper” chromatin; (6) nuclear molding; (7) binucleation or multinucleation; (8) granular cytoplasm; (9) clean background; (10) bloody background; (11) low cellularity; (12) air drying effect; (13) background material (amyloid, colloid, and calcification). Two additional cytologic features that may cause diagnostic confusion, (14) visible nucleoli and (15) nuclear pseudoinclusion, were also included.

### 2.4. Statistical Analyses

The distribution of cytologic features and background demographics among the different staining methods and diagnostic categories was compared using Fisher’s exact test. Post hoc pairwise comparisons between any two methods/categories were further made when the overall test was statistically significant. The number of atypical features between the accurately categorized or incorrectly categorized specimens was compared using the non-parametric Mann–Whitney U test. At last, the ability of cytomorphologic scores to discriminate accurately categorized or incorrectly categorized specimens was assessed using receiver operating characteristic (ROC) curve analysis. The confidence interval of the ROC curve was constructed using DeLong’s test. The optimal cutoff of cytomorphologic scores was determined by the Youden index. All tests were two-tailed, and *p* < 0.05 was considered statistically significant. The ROC analysis was conducted using MedCalc Statistical Software version 13.1.2.0 (MedCalc Software, Ostend, Belgium; https://www.medcalc.org; 2014). Other data analyses were performed using SPSS 25 (IBM SPSS Inc, Armonk, NY, USA).

## 3. Results

### 3.1. Overview of Cytodiagnosis and Morphological Features among Three Sample Groups (Papanicolaou Stain, Romanowsky Stain, and H&E Stain on Conventional Smears)

Of the 168 enrolled cytologic specimens, a cytologic diagnosis was made on 167 satisfactory samples based on the TBSRTC system. All aspiration materials, except for one, provided adequate cellularity for evaluation, and 145 (86.3%) cases were highly cellular. There was only one unsatisfactory sample in the H&E group, which was excluded from the subsequent statistical analysis.

Of the 167 satisfactory cytologic samples, cytologic diagnoses of MTC and MTC-S were rendered in 88.6% of cases (*n* = 148). These 148 samples were composed of 72 Papanicolaou stains, 39 Romanowsky stains, and 37 H&E stains. The remaining nineteen cases (11.4%) were categorized as malignancy NOS in 1.2% (*n* = 2); FN/SFN in thirteen cases (7.8%); and AUS/FLUS in four cases (2.4%). The morphologic characteristics, including staining methods and cytodiagnoses of the 19 discordant cases, are summarized in Table 1.

### 3.2. Cytologic Parameters of Accurately Categorized Groups

A comparison of the cytologic features rendered on the Papanicolaou-, Romanowsky-, and H&E-stained slides is presented in Table 2 and Table 3. For statistical analysis, the assessed parameters were classified as cytomorphologic (Table 2) and background features (Table 3). Most of the cytomorphologic parameters of MTC did not significantly differ among the three different staining groups (Table 2). Dyshesive cells were relatively less frequent in H&E-stained smears (*p* < 0.05). Nuclear molding was more frequent in Romanowsky- than in Papanicolaou-stained slides (*p* < 0.05). Comparing background characteristics, background blood and colloid were more frequently observed in Romanowsky and H&E groups, respectively (*p* < 0.05). Air drying was more common in H&E- than in Papanicolaou- and Romanowsky-stained slides (*p* < 0.05) (Table 3).

### 3.3. Cytologic Parameters of Discordant Diagnosis Group

A comparison of the cytologic features rendered on the Papanicolaou-, Romanowsky-, and H&E-stained slides is presented in Tables S1 and S2. Overall, the background factors and the cytomorphologic parameters did not significantly differ among the three staining groups. In addition, the results are still not statistically significant after excluding the one H&E-stained unsatisfactory smear (data not shown).

**Figure 1:**
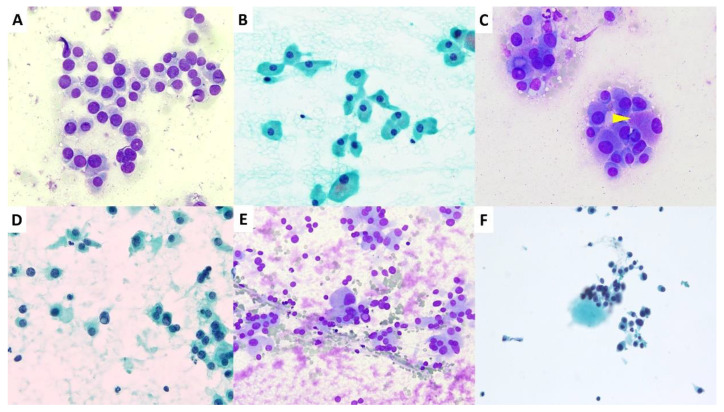
Cytomorphologic features of MTCs in the discordant group. (**A**) Round cells formed loosely cohesive clusters, making a diagnosis of FLUS or SFN (Romanowsky, ×400). (**B**,**C**) Polygonal tumor cells with abundant cytoplasm or cytoplasmic granules ((**C**); arrowhead), making a diagnosis of Hurthle cell neoplasm or FLUS ((**B**) Papanicolaou, ×400; (**C**) Liu, ×400). (**D**,**E**) Intranuclear cytoplasmic pseudoinclusion (center) mimicking papillary thyroid carcinoma ((**D**) Papanicolaou, ×400; (**E**): Liu, ×400). (**F**) A few plasmacytoid cells with preserved background colloid prompted an indeterminate diagnosis of AUS/FLUS (Papanicolaou, ×200).

In addition, Tables S3 and S4 summarize the frequency of cytomorphologic features in each diagnostic category. Regarding the cytomorphologic characteristics, the results demonstrate that high cellularity and polygonal cells were more frequently present in the FN/SFN and malignancy NOS groups than in the AUS/FLUS group (*p* < 0.05) (Figure 1A–C). Pseudoinclusions were also more frequently present in the malignancy NOS group than in the other two groups (*p* < 0.05) (Figure 1D,E). Nuclear molding was also more common in the AUS/FLUS and malignancy NOS groups than in the FN/SFN group (*p* < 0.05) (Table S3). Concerning background characteristics, low cellularity and air drying were more common in the AUS/FLUS group than in the FN/SFN group (*p* < 0.05) (Table S4). Our study reveals that all three discordant categories showed cellular pleomorphism and round cells, but they did exhibit some morphological differences (Figure 1A–F). 

### 3.4. Accurately Categorized Groups versus Discordant Diagnosis Groups

The morphologic features were compared between concordant cases and discordant cases. A comparison of the cytomorphologic parameters in all accurately categorized and discordant cases is presented in Table S5. Regarding the cytomorphologic characteristics, the results indicate that the round cells were more frequent in the incorrectly categorized specimens than the correctly categorized specimens (*p* < 0.05). However, the proportions of spindled cells, dyshesive cells, salt-and-pepper chromatin, and nuclear molding were significantly higher in the correctly categorized specimens than in the incorrectly categorized specimens (*p* < 0.05) (Table S5). Regarding the background characteristics, the results reveal that amyloid was more frequently present in the correctly categorized specimens than in the incorrectly categorized specimens (*p* < 0.05) (Table S6).

### 3.5. Minimal Cytologic Criteria for Diagnosing MTC

In our study, we summarized seven cytologic criteria that can be readily recognized by all three staining methods: high cellularity, cellular pleomorphism, plasmacytoid cells, round cells, dyshesive cells, salt-and-pepper chromatin, and binucleation or multinucleation. When the FNAs from the accurate and discordant cases were compared, an accurate diagnosis was achieved in 125 (84%) of the 148 samples whose FNAs exhibited 5 or more atypical features (Table 4). Nevertheless, 48% of discordant cases were found with three or four atypical features (*p* < 0.001). There was no difference in the scoring distribution among Papanicolaou, Romanowsky, and hematoxylin-eosin stains (*p* = 0.108) (data not shown).

**Figure 2:**
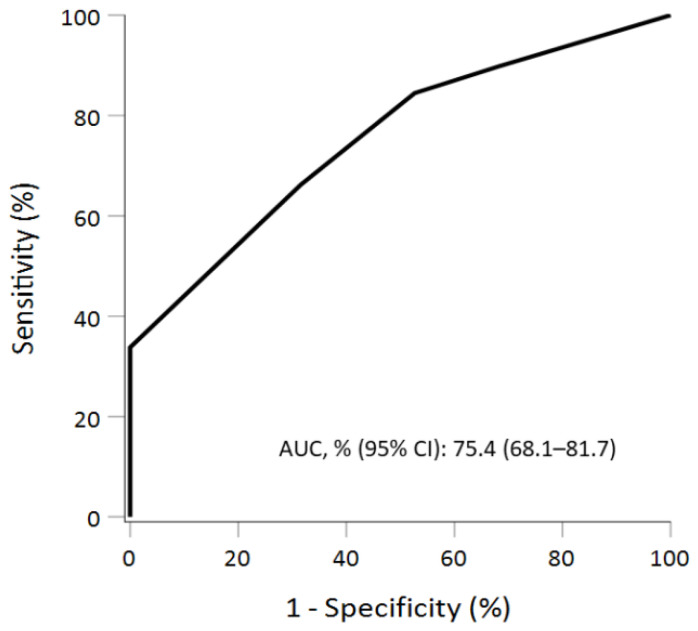
The receiver operating characteristic (ROC) curve of the cytomorphologic scoring. The performance of discrimination was satisfied, with an area under the ROC curve (AUC) of 75.4% (95% CI, 68.1–81.7%).

These observations laid the ground for a possible discrimination system based on cytomorphologic scores. A scoring system was proposed and applied to minimize misinterpreting errors using only cytologic variables. The receiver operating characteristic (ROC) curve of the scoring system is shown in Figure 2. The performance of discrimination for the scoring system was satisfied, with an area under the ROC curve (AUC) of 75.4% (95% CI, 68.1–81.7%). The corresponding diagnostic properties, including sensitivity and specificity, are also presented (Table 5). The optimal cutoff according to the Youden index was equal to or larger than 6, with a sensitivity of 66.2% (95% CI: 58–73.8%) and a specificity of 68.4% (95% CI: 43.4–87.4%).

## 4. Discussion

According to the previous studies by the Asian Thyroid Working Group, the thyroid FNA practices in the Asia-Pacific region vary from those of Western countries in multiple aspects [16,17,18,19,20,21]. Regarding the preparation of cytology smears, the Papanicolaou stain is the most widely used staining method for thyroid FNA specimens [22]. Most Chinese pathologists favored the H&E stain. In India, Taiwan, and Thailand, a combination of two stains: Papanicolaou stain for alcohol-fixed smears and Romanowsky stain for air-dried smears, has been used [22]. 

The incidence of MTC has been reported in recent studies. Miranda-Filho et al. executed a global assessment and comparison of incidence trends for major thyroid cancer [23]. Among the four surveyed Asian countries, the age-standardized incidence rates of MTC were in the range of 0.05–0.49 cases per 100,000 women and 0.08–0.41 cases per 100,000 men. The incidence rates were slightly lower than in Western countries (0.13–0.82 cases per 100,000 women and 0.07–0.62 cases per 100,000 men) [23]. In large case series from Asian countries, MTC accounted for 0.5% (Korea), 1.2% (Japan), 1.3% (China), and 2.8% (Taiwan) of all thyroid malignancies [4,24,25,26]. Our previous report introduced how MTC is handled in contemporary Asian thyroid FNA practice [14]. We also summarized that a high diagnostic accuracy could be achieved using the Romanowsky stain, Papanicolaou stain, and/or H&E stain. In addition, the cytologic features of MTC in conventional cytology have been well described [7,8,27,28,29,30,31]. However, to our knowledge, this study is the first to evaluate all of the cytologic features among different staining methods and the usefulness in the diagnosis of MTC.

The characteristic cytologic features of MTC are sometimes misleading. For example, dispersed cell patterns and an oxyphilic cytoplasm yielded the impression of follicular or oxyphilic neoplasm [32,33]. Recognition of the constitutive cytomorphologic features is needed for each cytopreparatory method, which may result in a lower threshold to initiate further workup for MTC.

### 4.1. Constitutive Cytomorphologic Features in Different Cytopreparatory Methods

According to Table 2, our study reveals that 7 out of the 11 cytologic parameters could be readily recognized by all three cytopreparatory methods (>50% frequency): high cellularity, cellular pleomorphism, plasmacytoid cells, round cells, dyshesive cells, salt-and-pepper chromatin, and binucleation or multinucleation. Nuclear molding was more apparent in the Romanowsky group. Dyshesive cells were seen at a remarkably high frequency on Papanicolaou and H&E smears. There was no significant difference found regarding polygonal cells, spindled cells, and granular cytoplasm.

Among the discordant cases, only two characteristic features (high cellularity and round cells) were consistently revealed in the three cytopreparatory methods (>50% frequency). Although the differences were not statistically significant, polygonal cells were relatively easily found on the Romanowsky stain. Visible nucleoli, intranuclear pseudoinclusions, and salt-and-pepper chromatin were comparably interpretable on the Papanicolaou stain and Romanowsky stain. 

There are several histologic variants of MTC including tubular (follicular), papillary, small cell, giant cell, clear cell, melanotic (pigmented), oncocytic (oxyphilic), squamous, amphicrine (composite calcitonin- and mucin-producing), and paraganglioma-like variants [1,2,3]. Therefore, difficulties exist in the differential diagnosis between MTC and follicular/oncocytic neoplasm, anaplastic carcinoma, papillary carcinomas, metastatic tumors, and even benign hyperplastic nodules [4,7,9,34,35]. In our study, most of the discordant aspirates were interpreted as FN/SFN and AUS/FLUS. These cases also presented with increased round cell populations, polygonal cells, and binucleated cells, together with an unobtrusive cytoplasm. In addition, the presence of visible nucleoli and nuclear pseudoinclusions also led to a misdiagnosis of follicular/Hurthle neoplasm or PTC (Figure 1). Cytoplasmic features are valuable in the diagnosis of MTC. On the Romanowsky- or Papanicolaou-stained slides, loose granularity of the cytoplasm was observed in MTC cells as opposed to the dense, firmly granular cytoplasm seen in oncocytic follicular neoplasm [33,36]. Azurophilic cytoplasmic granules (neurosecretory granules) seen on the Romanowsky stain also facilitate MTC diagnosis [33]. Therefore, a pattern-based and algorithmic approach to cytomorphologic features is mandatory for each cytopreparatory method. In the following section, we further analyze the association between the diagnostic performance and these significant atypical features.

### 4.2. Cytomorphologic Clues to Diagnose MTC on FNA

Papaparaskeva et al. reviewed the aspirates from 128 patients of MTC. The important cytologic criteria included a dispersed cell pattern of polygonal or triangular cells, azurophilic cytoplasmic granules, extremely eccentrically placed nuclei with coarsely granular chromatin, and the presence of amyloid, although none of the above can be used as a pathognomonic feature [8]. On the other hand, Dyhdalo et al. reported that expression of only one or none of the “classic” morphologic features of MTC (plasmacytoid or spindled cells, dyshesion, multinucleation, and salt-and-pepper chromatin) led to discrepancies in cytologic diagnosis [37]. This highlights a critical discrepancy in cytologic interpretation that can dramatically alter the performance of FNA. Therefore, we further focused on the discordant cases if all of the morphologic criteria were not demonstrated in different stains.

According to current practice, most discordant cases are frequently included in the category of indeterminate proliferations (FN/SFN and AUS/FLUS) due to either the lack of or the presence of subtle nuclear features of MTC, which do not allow for a definitive cytologic diagnosis of malignancy [9]. The cellular heterogeneity of MTCs may explain the misinterpretation based solely on traditional cytologic methods. Interobserver variability in the interpretation of the indeterminate cytologic categories is another likely contributing factor [38,39]. The interpretive discrepancies also reflect a need for concise diagnostic criteria with good observer agreement. 

Thus, we believe it necessary to consider a more objective evaluation using a unified scoring system. As previously mentioned, we summarized seven cytologic parameters for diagnosing MTC: high cellularity, cellular pleomorphism, plasmacytoid cells, round cells, dyshesive cells, salt-and-pepper chromatin, and binucleation or multinucleation, easily recognized regardless of the cytopreparatory method. To the best of our knowledge, the scoring system we have described herein is the first model that can differentiate MTC from misinterpreted results using only cytologic variables. A total score of 6 or 7 warrants a diagnosis of MTC/MTC-S based on the TBSRTC system. This proposed scoring system is applicable to minimizing misinterpreting errors and further improving the diagnostic accuracy of FNA.

Our study has a few limitations. Being a retrospective study, it is likely to have an inherent bias, potentially affecting the quality of data acquisition. Secondly, since it is a multi-institutional study, despite the application of consistent diagnostic criteria (i.e., TBSRTC), interobserver variation cannot be removed. Additionally, because of the retrospective design, only MTC patients were included in the study cohort. An external validation cohort including various diagnostic settings is required to evaluate the feasibility of our scoring system. In the current study, the statistical analysis was based on the performance of FNA among MTC patients. The sensitivity or specificity for general populations cannot be revealed due to the limitation of the study cohort. 

## 5. Conclusions

Our study evaluated the constitutive cytomorphologic features of MTC using different staining methods and combined these diagnostic parameters into a scoring system. In our approach, the presence of six or more atypical features achieves a desirable accuracy. Our easy-to-use scoring system could be helpful to identify patients at high risk for MTC preoperatively. These notable cytomorphologic clues should facilitate conclusive ancillary testing and clinical management.

## Figures and Tables

**Table 1:** Cytopathological characteristics of all the cytologic specimens with incorrect diagnoses (excluding unsatisfactory specimen).

No.	Staining Method	Cytologic Diagnosis	High Cellularity	Cellular Pleomorphism	Plasmacytoid Cells	Round Cells	Polygonal Cells	Spindled Cells	Dyshesive Cells	Salt-and-Pepper Chromatin	Pseudoinclusion	Visible Nucleoli	Nuclear Molding	Granular Cytoplasm	Binucleation or Multinucleation
1	Pap	Malignancy, NOS	Yes	Yes	Yes	Yes	Yes	No	Yes	No	Yes	No	No	Yes	Yes
2	Pap	Malignancy, NOS	Yes	Yes	No	Yes	Yes	No	Yes	Yes	Yes	No	Yes	Yes	Yes
3	Pap	AUS/FLUS	No	Yes	Yes	Yes	No	No	No	Yes	No	No	No	Yes	Yes
4	H&E	FN/SFN	Yes	No	Yes	Yes	No	No	No	No	No	No	No	No	No
5	Pap	FN/SFN	Yes	No	No	Yes	Yes	No	No	Yes	No	No	No	No	No
6	Rom	FN/SFN	Yes	No	Yes	Yes	Yes	No	No	No	No	No	No	Yes	Yes
7	Pap	FN/SFN	Yes	Yes	No	Yes	No	No	No	No	No	Yes	No	Yes	No
8	Rom	FN/SFN	Yes	Yes	No	Yes	Yes	No	No	No	No	No	No	Yes	Yes
9	Pap	FN/SFN	Yes	Yes	Yes	Yes	Yes	No	No	No	No	Yes	No	Yes	Yes
10	Rom	FN/SFN	Yes	Yes	Yes	Yes	Yes	No	Yes	No	No	No	No	No	Yes
11	Pap	FN/SFN	Yes	Yes	No	Yes	Yes	No	Yes	Yes	No	No	No	No	Yes
12	Rom	FN/SFN	Yes	Yes	Yes	Yes	Yes	No	Yes	No	No	No	No	No	Yes
13	Pap	FN/SFN	Yes	Yes	No	Yes	No	Yes	Yes	Yes	No	No	No	No	Yes
14	Rom	FN/SFN	Yes	Yes	Yes	Yes	Yes	Yes	No	No	No	No	No	No	Yes
15	Pap	FN/SFN	Yes	Yes	Yes	Yes	Yes	Yes	No	No	No	No	No	No	Yes
16	Rom	FN/SFN	Yes	Yes	No	Yes	Yes	Yes	No	No	No	No	No	Yes	Yes
17	Pap	AUS/FLUS	No	Yes	No	Yes	No	No	No	Yes	No	No	Yes	No	No
18	Rom	AUS/FLUS	No	Yes	No	Yes	No	No	No	Yes	No	No	Yes	No	No
19	Pap	AUS/FLUS	No	Yes	Yes	Yes	No	No	No	No	No	No	No	Yes	No

Pap: Papanicolaou stain; H&E: hematoxylin and eosin; Rom: Romanowsky staining; NOS: not otherwise specified; AUS/FLUS: atypia of undetermined significance or follicular lesion of undetermined significance; FN: follicular neoplasm; SFN: suspicious for a follicular neoplasm.

**Table 2:** Cytomorphologic characteristics by different staining methods (accurately categorized specimens, total *n* = 148).

Parameter	Papanicolaou Stain (*n* = 72)	Romanowsky Stain (*n* = 39)	H&E Stain (*n* = 37)	*p*-Value #
High cellularity	62 (86%)	35 (90%)	31 (84%)	0.740
Cellular pleomorphism	64 (89%)	33 (85%)	32 (86%)	0.812
Plasmacytoid cells	53 (74%)	28 (72%)	25 (68%)	0.770
Round cells	58 (81%)	24 (62%)	25 (68%)	0.074
Polygonal cells	43 (60%)	21 (54%)	18 (49%)	0.522
Spindled cells	33 (46%)	17 (44%)	20 (54%)	0.624
Dyshesive cells	70 (97%)	39 (100%)	31 (84%) ^a,b^	0.006
Salt-and-pepper chromatin	67 (93%)	38 (97%)	36 (97%)	0.684
Pseudoinclusion	18 (25%)	11 (28%)	17 (46%)	0.085
Visible nucleoli	6 (8%)	3 (8%)	0 (0%)	0.210
Nuclear molding	32 (44%)	27 (69%) ^a^	22 (59%)	0.036
Granular cytoplasm	20 (28%)	12 (31%)	13 (35%)	0.736
Binucleation/multinucleation	50 (69%)	30 (77%)	20 (54%)	0.098

# Fisher’s exact test; ^a^: *p* < 0.05 versus Papanicolaou; ^b^: *p* < 0.05 versus Romanowsky.

**Table 3:** Background characteristics by different staining methods (accurately categorized specimens, total *n* = 148).

Parameter	Papanicolaou Stain (*n* = 72)	Romanowsky Stain (*n* = 39)	H&E Stain (*n* = 37)	*p*-Value #
Clean	51 (71%)	23 (59%)	29 (78%)	0.185
Bloody	22 (31%)	27 (69%) ^a^	9 (24%) ^b^	<0.001
Low cellularity	10 (14%)	4 (10%)	6 (16%)	0.740
Air drying	27 (38%)	11 (28%)	1 (3%) ^a,b^	<0.001
Amyloid	33 (46%)	22 (56%)	16 (43%)	0.446
Colloid	12 (17%)	5 (13%)	17 (46%) ^a,b^	0.001
Calcification	3 (4%)	2 (5%)	2 (5%)	1.000

# Fisher’s exact test; ^a^: *p* < 0.05 versus Papanicolaou; ^b^: *p* < 0.05 versus Romanowsky.

**Table 4:** The comparison of the presence of significant cytologic features between accurately and incorrectly diagnosed cases of MTC.

Cytological Features Seen in Smears	Accurately Categorized (*n* = 148)	Incorrectly Categorized (*n* = 19)	*p*-Value #
All 7 features present	50 (34%)	0 (0%)	<0.001
Any 6 of the 7 features present	48 (32%)	6 (32%)	
Any 5 of the 7 features present	27 (18%)	4 (21%)	
Any 4 of the 7 features present	8 (5%)	3 (16%)	
Any 3 of the 7 features present	15 (10%)	6 (32%)	
Any 2 of the 7 features present	0 (0%)	0 (0%)	
Any 1 of the 7 features present	0 (0%)	0 (0%)	
None of the 7 features present	0 (0%)	0 (0%)	

# Mann–Whitney U test.

**Table 5:** Diagnostic property of significant cytologic features in FNA for discriminating accurately categorized or incorrectly categorized specimens.

Cytologic Features in FNA	Sensitivity, % (95% CI)	Specificity, % (95% CI)
≥4	89.9 (83.8–94.2)	31.6 (12.6–56.6)
≥5	84.5 (77.6–89.9)	47.4 (24.4–71.1)
≥6 *	66.2 (58.0–73.8)	68.4 (43.4–87.4)
≥7	33.8 (26.2–42.0)	100.0 (82.4–100.0)

FNA: fine-needle aspiration; CI: confidence interval; * the optimal cutoff by the Youden index.

## Data Availability

Data are available on request due to all institutional restrictions related to patient privacy.

## Supplementary Materials

The following are available online at https://www.mdpi.com/article/10.3390/diagnostics11081396/s1.

## References

[1] DeLellis R.A., Al Ghuzlan A., Albores Saavedra J., Baloch Z.W., Basolo F., Elisei R., Kaserer K., LiVolsi V., Matias-Guiu X., Mete O., Lloyd R.V., Osamura R.Y., Klöpel G., Rosai J. (2017). Medullary thyroid carcinoma. WHO Classification of Tumours of Endocrine Organs.

[2] Bugalho M.J., Santos J.R., Sobrinho L. (2005). Preoperative diagnosis of medullary thyroid carcinoma: Fine needle aspiration cytology as compared with serum calcitonin measurement. J. Surg. Oncol..

[3] Simoes-Pereira J., Bugalho M.J., Limbert E., Leite V. (2016). Retrospective analysis of 140 cases of medullary thyroid carcinoma followed-up in a single institution. Oncol. Lett..

[4] Suzuki A., Hirokawa M., Takada N., Higuchi M., Ito A., Yamao N., Hayashi T., Kuma S., Miyauchi A. (2017). Fine-needle aspiration cytology for medullary thyroid carcinoma: A single institutional experience in Japan. Endocr. J..

[5] Trimboli P., Treglia G., Guidobaldi L., Romanelli F., Nigri G., Valabrega S., Sadeghi R., Crescenzi A., Faquin W.C., Bongiovanni M. (2015). Detection rate of FNA cytology in medullary thyroid carcinoma: A meta-analysis. Clin. Endocrinol..

[6] Essig G.F., Porter K., Schneider D., Debora A., Lindsey S.C., Busonero G., Fineberg D., Fruci B., Boelaert K., Smit J.W. (2013). Fine needle aspiration and medullary thyroid carcinoma: The risk of inadequate preoperative evaluation and initial surgery when relying upon FNAB cytology alone. Endocr. Pract..

[7] Forrest C.H., Frost F.A., de Boer W.B., Spagnolo D.V., Whitaker D., Sterrett B.F. (1998). Medullary carcinoma of the thyroid: Accuracy of diagnosis of fine-needle aspiration cytology. Cancer.

[8] Papaparaskeva K., Nagel H., Droese M. (2000). Cytologic diagnosis of medullary carcinoma of the thyroid gland. Diagn. Cytopathol..

[9] Ali S.Z., Cibas E.S. (2017). The 2017 Bethesda System for Reporting Thyroid Cytopathology. J. Am. Soc. Cytopathol..

[10] Thomas C.M., Asa S.L., Ezzat S., Sawka A.M., Goldstein D. (2019). Diagnosis and pathologic characteristics of medullary thyroid carcinoma-review of current guidelines. Curr. Oncol..

[11] Kihara M., Hirokawa M., Kudo T., Hayashi T., Yamamoto M., Masuoka H., Higashiyama T., Fukushima M., Ito Y., Miya A. (2018). Calcitonin measurement in fine-needle aspirate washout fluid by electrochemiluminescence immunoassay for thyroid tumors. Thyroid Res..

[12] Trimboli P., Guidobaldi L., Bongiovanni M., Crescenzi A., Alevizaki M., Giovanella L. (2016). Use of fine-needle aspirate calcitonin to detect medullary thyroid carcinoma: A systematic review. Diagn. Cytopathol..

[13] Trimboli P., Giovanella L., Crescenzi A., Romanelli F., Valabrega S., Spriano G., Cremonini N., Guglielmi R., Papini E. (2014). Medullary thyroid cancer diagnosis: An appraisal. Head Neck.

[14] Liu C.Y., Bychkov A., Agarwal S., Zhu Y., Hang J.F., Lai C.R., Na H.Y., Li W., Liu Z., Jain D. (2021). Cytologic diagnosis of medullary thyroid carcinoma in the Asia-Pacific region. Diagn. Cytopathol..

[15] Bychkov A., Kakudo K., Hong S. (2017). Current Practices of Thyroid Fine-Needle Aspiration in Asia: A Missing Voice. J. Pathol. Transl. Med..

[16] Kakudo K., Higuchi M., Hirokawa M., Satoh S., Jung C.K., Bychkov A. (2017). Thyroid FNA cytology in Asian practice-Active surveillance for indeterminate thyroid nodules reduces overtreatment of thyroid carcinomas. Cytopathology.

[17] Agarwal S., Bychkov A., Jung C.K., Hirokawa M., Lai C.R., Hong S., Kwon H.J., Rangdaeng S., Liu Z., Su P. (2019). The prevalence and surgical outcomes of Hürthle cell lesions in FNAs of the thyroid: A multi-institutional study in 6 Asian countries. Cancer Cytopathol..

[18] Kakudo K., Bychkov A., Abelardo A., Keelawat S., Kumarasinghe P. (2019). Malpractice Climate Is a Key Difference in Thyroid Pathology Practice Between North America and the Rest of the World. Arch. Pathol. Lab. Med..

[19] Vuong H.G., Ngo H., Bychkov A., Jung C.K., Vu T.H., Lu K.B., Kakudo K., Kondo T. (2020). Differences in surgical resection rate and risk of malignancy in thyroid cytopathology practice between Western and Asian countries: A systematic review and meta-analysis. Cancer Cytopathol..

[20] Rashid F.A., Munkhdelger J., Fukuoka J., Bychkov A. (2020). Prevalence of *BRAFV600E* mutation in Asian series of papillary thyroid carcinoma-a contemporary systematic review. Gland Surg..

[21] Vuong H.G., Suzuki A., Na H.Y., Tuyen P.V., Khuy D.M., Nguyen H.C., Jitpasutham T., Abelardo A., Amano T., Park S.Y. (2021). Application of the Bethesda System for Reporting Thyroid Cytopathology in the Pediatric Population. Am. J. Clin. Pathol..

[22] Jung C.K., Hong S., Bychkov A., Kakudo K. (2017). The Use of Fine-Needle Aspiration (FNA) Cytology in Patients with Thyroid Nodules in Asia: A Brief Overview of Studies from the Working Group of Asian Thyroid FNA Cytology. J. Pathol. Transl. Med..

[23] Miranda-Filho A., Lortet-Tieulent J., Bray F., Cao B., Franceschi S., Vaccarella S., Dal Maso L. (2021). Thyroid cancer incidence trends by histology in 25 countries: A population-based study. Lancet Diabetes Endocrinol..

[24] Ahn H.Y., Chae J.E., Moon H., Noh J., Park Y.J., Kim S.G. (2020). Trends in the Diagnosis and Treatment of Patients with Medullary Thyroid Carcinoma in Korea. Endocrinol. Metab..

[25] Du L., Wang Y., Sun X., Li H., Geng X., Ge M., Zhu Y. (2018). Thyroid cancer: Trends in incidence, mortality and clinical-pathological patterns in Zhejiang Province, Southeast China. BMC Cancer.

[26] Lin J.D., Weng H.F., Huang M.J., Huang B.Y., Huang H.S., Jeng L.B. (1994). Thyroid cancer treated in Chang Gung Memorial Hospital (northern Taiwan) during the period 1979–1992: Clinical presentation, pathological finding, analysis of prognostic variables, and results of treatment. J. Surg. Oncol..

[27] Lertprasertsuke N., Kakudo K., Nakamura A., Tada N., Osamura Y., Watanabe K., Kubota M. (1989). C cell carcinoma of the thyroid. Follicular variant. Acta Pathol. Jpn..

[28] Harach H.R., Bergholm U. (1988). Medullary (C cell) carcinoma of the thyroid with features of follicular oxyphilic cell tumours. Histopathology.

[29] Schaffer R., Muller H.A., Pfeifer U., Ormanns W. (1984). Cytological findings in medullary carcinoma of the thyroid. Pathol. Res. Pract..

[30] Kakudo K., Miyauchi A., Takai S., Katayama S., Kuma K., Kitamura H. (1979). C cell carcinoma of the thyroid—Papillary type. Acta Pathol. Jpn..

[31] Kakudo K., Miyauchi A., Ogihara T., Takai S.I., Kitamura H., Kosaki G., Kumahara Y. (1978). Medullary carcinoma of the thyroid. Giant cell type. Arch. Pathol. Lab. Med..

[32] Agarwal C., Raychaudhuri S., Batra A., Pujani M., Dhingra S. (2019). Medullary carcinoma of thyroid mimicking Hurthle cell neoplasm on cytology: A diagnostic dilemma. Diagn. Cytopathol..

[33] Sams S.B., Tompkins K.D., Mayson S., Raeburn C.D., Mehrotra S. (2017). Oncocytic variant of medullary thyroid carcinoma; a rare tumor with numerous diagnostic mimics by fine needle aspiration. Diagn. Cytopathol..

[34] Buzdugă C.M., Costea C.F., Cărăuleanu A., Lozneanu L., Turliuc M.D., Cucu A.I., Ciocoiu M., Floria M., Tănase D.M., Dragomir R.A. (2019). Protean cytological, histological and immunohistochemical appearances of medullary thyroid carcinoma: Current updates. Rom. J. Morphol. Embryol..

[35] Zhu Y., Wang T., Kakudo K. (2019). Medullary (C Cell) Thyroid Carcinoma or Oxyphilic Follicular Neoplasms. Thyroid FNA Cytology.

[36] Montone K.T., Baloch Z.W., LiVolsi V.A. (2008). The thyroid Hurthle (oncocytic) cell and its associated pathologic conditions: A surgical pathology and cytopathology review. Arch. Pathol. Lab. Med..

[37] Dyhdalo K.S., Chute D.J. (2018). Barriers to the recognition of medullary thyroid carcinoma on FNA: Implications relevant to the new American Thyroid Association guidelines. Cancer Cytopathol..

[38] Cibas E.S., Baloch Z.W., Fellegara G., LiVolsi V.A., Raab S.S., Rosai J., Diggans J., Friedman L., Kennedy G.C., Kloos R.T. (2013). A prospective assessment defining the limitations of thyroid nodule pathologic evaluation. Ann. Intern. Med..

[39] Thodou E., Canberk S., Schmitt F. (2021). Challenges in Cytology Specimens with Hürthle Cells. Front. Endocrinol..

