# Supplementary material for: Constitutive Cytomorphologic Features of Medullary Thyroid Carcinoma Using Different Staining Methods
Source: Diagnostics (Basel). 2021 Aug 2;11(8):1396. doi: 10.3390/diagnostics11081396 (PMC8392035; doi:10.3390/diagnostics11081396)
Supplement: Supplementary file 1 [file diagnostics-11-01396-s001.zip › diagnostics-1310697-supplementary.pdf]

Table S1. Cytomorphologic characteristics by different staining methods (excluding unsatisfactory specimen)(total n=19)

Parameter/Stain	Papanicolaou stain (n = 11)	Romanowsky stain (n = 7)	H&E stain (n = 1)	*P*-value#
High cellularity	9 (82%)	7 (100%)	1 (100%)	0.550
Cellular pleomorphism	10 (91%)	6 (86%)	0 (0%)	0.194
Plasmacytoid cells	5 (45%)	4 (57%)	1 (100%)	1.000
Round cells	11 (100%)	7 (100%)	1 (100%)	-
Polygonal cells	6 (55%)	6 (86%)	0 (0%)	0.211
Spindled cells	2 (18%)	2 (29%)	0 (0%)	1.000
Dyshesive cells	4 (36%)	2 (29%)	0 (0%)	1.000
Salt-and-pepper chromatin	6 (55%)	1 (14%)	0 (0%)	0.211
Pseudoinclusion	2 (18%)	0 (0%)	0 (0%)	0.550
Visible nucleoli	2 (18%)	0 (0%)	0 (0%)	0.550
Nuclear molding	2 (18%)	1 (14%)	1 (100%)	0.404
Granular cytoplasm	6 (55%)	3 (43%)	0 (0%)	1.000
Binucleation or multinucleation	7 (64%)	6 (86%)	0 (0%)	0.285

Fisher's exact test.

Table S2. Background characteristics by different staining methods (excluding unsatisfactory specimen)(total n=19)

Parameter/Stain	Papanicolaou stain (n = 11)	Romanowsky stain (n = 7)	H&E stain (n = 1)	*P*-value#
Clean	5 (45%)	4 (57%)	0 (0%)	1.000
Bloody	6 (55%)	3 (43%)	1 (100%)	1.000
Low cellularity	2 (18%)	1 (14%)	0 (0%)	1.000
Air drying	1 (9%)	1 (14%)	0 (0%)	1.000
Amyloid	2 (18%)	1 (14%)	0 (0%)	1.000
Colloid	3 (27%)	3 (43%)	0 (0%)	0.745
Calcification	0 (0%)	0 (0%)	0 (0%)	-

Fisher's exact test.

Table S3. Cytomorphologic characteristics by different cytologic diagnoses (exclude unsatisfactory specimen)(total n=19)

Parameter/Diagnosis	FN/FN-H/SFN (n = 13)	AUS/FLUS (n = 4)	Malignancy, NOS (n = 2)	*P*-value#
High cellularity	13 (100%)	0 (0%)[a]	2 (100%)[b]	**<0.001**
Cellular pleomorphism	10 (77%)	4 (100%)	2 (100%)	0.678
Plasmacytoid cells	7 (54%)	2 (50%)	1 (50%)	1.000
Round cells	13 (100%)	4 (100%)	2 (100%)	-
Polygonal cells	10 (77%)	0 (0%)[a]	2 (100%)[b]	**0.009**
Spindled cells	4 (31%)	0 (0%)	0 (0%)	0.705
Dyshesive cells	4 (31%)	0 (0%)	2 (100%)	0.072
Salt-and-pepper chromatin	3 (23%)	3 (75%)	1 (50%)	0.154
Pseudoinclusion	0 (0%)	0 (0%)	2 (100%)[a,b]	**0.006**
Visible nucleoli	2 (15%)	0 (0%)	0 (0%)	1.000
Nuclear molding	0 (0%)	2 (50%)[a]	1 (50%)[a]	**0.021**
Granular cytoplasm	5 (38%)	2 (50%)	2 (100%)	0.406
Binucleation or multinucleation	10 (77%)	1 (25%)	2 (100%)	0.157

Fisher's exact test; a: $p<0.05$ versus FN/FN-H/SFN; b: $p<0.05$ versus AUS/FLUS.

Table S4. Background characteristics by different cytologic diagnoses (exclude unsatisfactory specimen)(total n=19)

Parameter/Diagnosis	FN/FN-H/SFN (n = 13)	AUS/FLUS (n = 4)	Malignancy, NOS (n = 2)	*P*-value#
Clean	5 (38%)	2 (50%)	2 (100%)	0.406
Bloody	8 (62%)	2 (50%)	0 (0%)	0.406
Low cellularity	0 (0%)	3 (75%)[a]	0 (0%)	**0.008**
Air drying	0 (0%)	2 (50%)[a]	0 (0%)	**0.041**
Amyloid	1 (8%)	2 (50%)	0 (0%)	0.115
Colloid	3 (23%)	3 (75%)	0 (0%)	0.157
Calcification	0 (0%)	0 (0%)	0 (0%)	-

Fisher's exact test; a: $p<0.05$ versus FN/FN-H/SFN.

Table S5. The comparison of cytomorphologic characteristics between accurately and incorrectly categorized cases

Parameter	Accurately categorized (n = 148)	Incorrectly categorized (n = 19)	*P*-value#
High cellularity	128 (86%)	17 (89%)	1.000
Cellular pleomorphism	129 (87%)	16 (84%)	0.720
Plasmacytoid cells	106 (72%)	10 (53%)	0.113
Round cells	107 (72%)	19 (100%)	**0.004**
Polygonal cells	82 (55%)	12 (63%)	0.627
Spindled cells	70 (47%)	4 (21%)	**0.047**
Dyshesive cells	140 (95%)	6 (32%)	**<0.001**
Salt-and-pepper chromatin	141 (95%)	7 (37%)	**<0.001**
Pseudoinclusion	46 (31%)	2 (11%)	0.103
Visible nucleoli	6 (4%)	2 (11%)	0.227
Nuclear molding	81 (55%)	4 (21%)	**0.007**
Granular cytoplasm	45 (30%)	9 (47%)	0.191
Binucleation or multinucleation	100 (68%)	13 (68%)	1.000

Fisher's exact test.

Table S6. The comparison of background characteristics between accurately and incorrectly categorized cases

Parameter	Accurately categorized (n = 148)	Incorrectly categorized (n = 19)	*P*-value#
Clean	103 (70%)	9 (47%)	0.069
Bloody	58 (39%)	10 (53%)	0.323
Low cellularity	20 (14%)	3 (16%)	0.729
Air drying	39 (24%)	2 (11%)	0.164
Amyloid	71 (48%)	3 (16%)	**0.012**
Colloid	34 (23%)	6 (22%)	0.402
Calcification	7 (5%)	0 (0%)	1.000

Fisher's exact test.
